# Back to the Salt Mines: Genome and Transcriptome Comparisons of the Halophilic Fungus *Aspergillus salisburgensis* and Its Halotolerant Relative *Aspergillus sclerotialis*

**DOI:** 10.3390/genes10050381

**Published:** 2019-05-20

**Authors:** Hakim Tafer, Caroline Poyntner, Ksenija Lopandic, Katja Sterflinger, Guadalupe Piñar

**Affiliations:** VIBT EQ Extremophile Center, Department of Biotechnology, University of Natural Resources and Life Sciences, Muthgasse 18, 1190 Vienna, Austria; hakim.tafer@boku.ac.at (H.T.); ksenija.lopandic@boku.ac.at (K.L.); katja.sterflinger@boku.ac.at (K.S.); guadalupe.pinar@boku.ac.at (G.P.) Correspondence: caroline.poyntner@boku.ac.at

**Keywords:** halophiles, halotolerant, fungi, transcriptomics, genomics

## Abstract

Salt mines are among the most extreme environments as they combine darkness, low nutrient availability, and hypersaline conditions. Based on comparative genomics and transcriptomics, we describe in this work the adaptive strategies of the true halophilic fungus *Aspergillus salisburgensis*, found in a salt mine in Austria, and compare this strain to the ex-type halotolerant fungal strain *Aspergillus sclerotialis*. On a genomic level, *A. salisburgensis* exhibits a reduced genome size compared to *A. sclerotialis*, as well as a contraction of genes involved in transport processes. The proteome of *A. sclerotialis* exhibits an increased proportion of alanine, glycine, and proline compared to the proteome of non-halophilic species. Transcriptome analyses of both strains growing at 5% and 20% NaCl show that *A. salisburgensis* regulates three-times fewer genes than *A. sclerotialis* in order to adapt to the higher salt concentration. In *A. sclerotialis*, the increased osmotic stress impacted processes related to translation, transcription, transport, and energy. In contrast, membrane-related and lignolytic proteins were significantly affected in *A. salisburgensis*.

## 1. Introduction

Saline and hypersaline environments like salt marshes, saline soil, and salt water, as well as the Dead Sea harbor a diverse community of fungi [1]. Most of these fungi do not require salt for growth and have optimum growth in the absence of salt. Nevertheless, they are halotolerant and can withstand a salt concentration up to 30% [2].

Real halophilic microorganisms are adapted to conditions of high salinity and require a certain concentration of NaCl for their optimum growth. Halophiles are phylogenetically quite diverse and can be found in the domains of Archaea, Bacteria, and Eukarya. Until almost a decade ago, it was a general belief in mycology and in food microbiology that fungi growing on substrates with low water activity (a_w_) have a general xerophilic phenotype [3] that is determined by the water potential of the medium, rather than by the chemical nature of the solute [4,5]. Therefore, fungi were considered xerophilic if they grew well at an a_w_ of 0.85, corresponding to 17% NaCl or 50% glucose added to their growth medium. Xerophilic Aspergilli, together with Penicillia, dominate the actively-growing mycoflora on dried food and also on materials stored in museums and archives. *Aspergillus vitricola* was first isolated from glass surfaces [6] and occurs frequently in house dust. Furthermore, *Aspergillus glabripes* occurs on books and archive material, as well as in house dust [7]. Xerophilic halotolerant Aspergilli were also shown to be common invaders of pipe organs in churches [8].

The term “halophile” for fungi was introduced in 1975 for those few xerophilic food-borne species that exhibit quite superior growth on media with NaCl as the controlling solute. Fungi have subsequently been described in moderately-saline environments, such as salt marshes, saline soil, and sea water, but were considered to be unable to grow in highly saline waters [2]. In 2000, fungi were isolated for the first time from the brine of solar salterns [2,9]. However, halophilic fungi are uncommon [2]. Currently, only *Wallemia ichthyophaga*, *Wallemia muriae*, *Aspergillus baarnensis*, *Aspergillus salinarium*, and *Aspergillus ruber* [10] are classified as halophiles. Recently, a fungus isolated from a historical wooden staircase in a salt mine in Austria was described as a new species, *Aspergillus saliburgiensis* [11,12], and was added to the list of halophilic fungi.

Cells living in natural saline systems, where high salt amounts cause high osmotic pressure, must maintain lower water potential than their surroundings in order to survive and proliferate. The ability to survive osmotic stress requires several adaptations. *Wallemia ichthyophaga* withstands high salt concentration by increasing the intracellular concentration of polyols, using high-affinity K${}^{+}$ transporters and increasing the thickness of its cell-wall by a factor of three [13]. Under high salt concentration, *Aspergillus ruber* increases the number of ion transporters, exhibits a higher proportion of acidic amino acids, restructures its cell wall, and uses glycerol as a compatible solute [10]. Similar adaptation strategies are found in halotolerant species, where the production of hydrophilic compounds, such as amino acids, sugar alcohols, and soluble sugars, have been reported [14,15,16,17,18].

The mechanisms behind the adaptive capacities of halotolerant and halophilic fungi have only begun to be understood in recent years, with the first study of the transcriptome of *Wallemia ichthyophaga* dating from 2013 [13]. Since then, an additional halophile, *Aspergillus ruber*, has been published [10]. In order to extend the knowledge in the field, we present the results of the comparative sequencing of the genomes of two species, *A. salisburgensis*, a halophilic fungus from a salt mine, and *A. sclerotialis*, as a representative of halotolerant fungi. Additionally, the two species were exposed to two concentrations of salt (5% and 20%), and the cellular response was studied on the transcriptome level. The genetic content, as well as the transcriptome, were compared to other halophilic, halotolerant, and to other well-studied, less salt-tolerant fungi (named as control) in order to better understand which mechanisms were involved in the resistance against osmotic stress.

## 2. Materials and Methods

### 2.1. Strains, Media, and Cultivation

The strain *Aspergillus salisburgensis* (EXF-10247/MA6005) was isolated in a previous study from a wooden staircase, built in the year 1108 B.C. and discovered in 2003 in a salt mine in the Austrian region “Salzkammergut”, Upper Austria [12]. The strain *A. sclerotialis* (Strain No. MA 5985, CBS 366.77) was supplied by the Fungal Biodiversity Centre (CBS), Utrecht, Netherlands. The two examined strains were grown on salt glucose media for the whole genome sequencing (5% glucose, 0.1% peptone, 0.1% malt extract, agar, and either 5% or 20% NaCl) and salt yeast media for the transcriptome sequencing (0.4% glucose, 0.4% yeast extract, 1.0% malt extract, 1.2% agar, and either 5% or 20 % NaCl). The two strains were incubated at their optimal temperature (*A. salisburgensis* at room temperature, *A. sclerotialis* at 37 ${}^{\circ }$C) for 35 days.

### 2.2. Whole Genome Sequencing

Genomic DNA extraction of pure fungal strains was performed as previously described [19]. Approximately one cm${}^{2}$ of fungal colonies, consisting of both hyphal and conidiogenous structures, were collected and placed in a 1.5-mL bead beater tube with 0.2-g glass beads (0.75–1 mm, Carl Roth GmbH Co., KG, Karlsruhe, Germany) and 500 μL of CTAB-buffer (1.2 g Tris-HCl, 8.2 g NaCl, 0.81 g EDTA × 2 H_2_O, 2.0 g CTAB, and 0.2 g β-mercaptoethanol, pH 8.0) and processed twice in the Fast Prep FP120 Ribolyzer (Thermo Savant, Holbrook, AZ, USA) for 40 s at a speed of 4 m/s. Between these ribolyzing steps, samples were incubated at 65 ${}^{\circ }$C for 10 min at 300 rpm. Further DNA extraction was performed with phenol/chloroform/isoamyl alcohol. Genome sequencing was carried out using the ION Proton Technology (Ion AmpliSeq Library Preparation kit, Template OT2 200 kit, Ion PI Sequencing 200 kit, Ion PI chip kit V2, Life Technologies, Carlsbad, CA, USA) following the instructions of the manufacturers.

### 2.3. Total RNA Extraction

After 35 days of cultivation, colonies were collected as described in Section 2.2, and RNA was extracted from the biomass using the FastRNA Pro RED Kit (MP Biomedicals, Santa Ana, CA, USA). For each condition and strain, three replicates of total RNA were extracted following the manufacture’s protocol. The quality and quantity was measured using the Agilent 2100 Bioanalyzer (Agilent Technologies, Santa Clara, CA, USA) and a Qubit 2.0 (Life Technologies, Carlsbad, CA, USA).

### 2.4. RNA Library Preparation

The total RNA library was prepared as described before [20]. mRNA was isolated from the total RNA using the Dynabeads mRNA DIRECT Micro Kit (Ambion, Life Technologies, Carlsbad, CA, USA). Subsequently, the RNA library for sequencing was constructed using the Ion Total RNA-Seq kit v2 (Life Technologies, Carlsbad, CA, USA). Quality was checked using the Agilent 2100 Bioanalyzer instrument (Agilent Technologies, Santa Clara, CA, USA). The final library was size selected to 290 bp using Pippin Prep (Sage Science, Beverly, MA, USA). The sequencing was done using the Ion Torrent Chef instrument, Ion Torrent Proton instrument, and the HiQ sequencing kit (Life Technologies, Carlsbad, CA, USA).

### 2.5. Genome Annotation and Assembly

*A. salisburgensis* and *A. sclerotialis* were assembled with Newbler. Genome completeness was assessed with BUSCO [21]. The final protein annotation was obtained by passing the ab initio protein annotation generated by BUSCO with the help of Augustus [22] to BRAKER2 [23,24] together with the RNA sequencing data. The functional annotation was done with InterProScan 5.25.64 [25] and EggNog [26] with default settings. Homologies with proteins in the Transporter Classification Database (TCDB) [27], the peptidase database (MEROPS) [28], and the Carbohydrate-Active Enzymes Database (CAZY) [29] were assessed with Blastp [30] (E-value <0.001).

### 2.6. Comparative Genomics

Protein orthology was assessed with ProteinOrtho 5.16b [31], where BLAST was replaced with DIAMOND [32]. The species phylogenomic tree was generated with iqtree [33,34,35] based on the MAFFT-alignment of one-to-one homologues. The TimeTree was inferred using the Reltime [36,37] method and ordinary least squares estimates of branch length. The TimeTree was computed using 8 calibration constraints retrieved from TimeTree.org. All position containing gaps and missing data were eliminated. There was a total of 37,607 positions in the final dataset. Evolutionary analyses were conducted in MEGA X [38].

Protein family expansion and contraction were computed with CAFE [39] and the previously-obtained TimeTree. The significance of protein families over- and under-representation was computed with the Fisher exact test.

Amino acids in the one-to-one conserved proteins and proteins containing a signal peptide, as annotated by SignalP [40], were tested for distribution bias with Wilcoxon tests in R [41].

### 2.7. Differential Expression

Genes’ differential expression was obtained with kallisto [42], tximport [43] and DeSeq2 [44]. All figures were made in R with ggplot2 [45], ggtree [46], and deeptime.

## 3. Results and Discussion

### 3.1. Highly Complete Genome and Gene Set

The genome assemblies of *A. salisburgensis* and *A. sclerotialis* used for this study were sequenced on the Ion Proton sequencing platform (Section 2.2, Section 2.3 and Section 2.4). The assembled genome of *A. salisburgensis* had a size of 21.9 Mb and contained a total of 8895 protein-coding loci for a total of 1351 contigs (>500 bp). *A. sclerotialis* had a 27% larger genome (27.9 Mb) with 27% more coding-genes (11307), compared to *A. salisburgensis*, and 1704 contigs (Supplementary Table S1). Among the 4406 highly-conserved genes in Eurohiomycetes [21], 96% were found in *A. sclerotialis* and 94% were present in *A. salisburgensis*. It was reported that compact genomes with a reduced number of protein-coding genes might be a characteristic of obligate halophiles, such as *Wallemia ichthyophaga* [15]. This trend could not be confirmed in Aspergilli where the genome of the halophile *Aspergillus ruber* [10] has a similar size and gene content compared to *A. sclerotialis*.

### 3.2. Gene Conservation

The gene conservation pattern between *A. salisburgensis*, *A. sclerotialis*, two halophiles (Table 1, marked as HH), six halotolerant (marked as H), and three control strains exhibiting no salt resistance (marked as C) were studied with ProteinOrtho [31].

**Figure 1:**
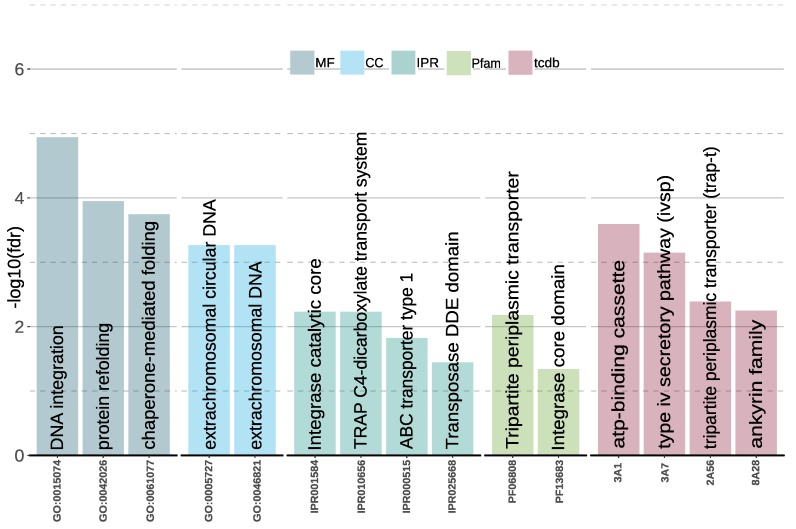
Graphical representation of the $-log_{10}\left(fdr\right)$ of the enriched categories in CAZY (Carbohydrate-Active Enzyme Database), Gene Ontology (GO), InterProScan (IPR), Protein Family (PFAM) and Transporter Classification Database (TCDB) in the set of *A. salisburgensis*-specific genes. Functional categories related to chitin degradation, protein folding, transport, and DNA integration are overrepresented.

ProteinOrtho identified 1169 groups of orthologs present in all 13 species, 1091 genes specific to *A. salisburgensis*, and 2362 genes specific to *A. sclerotialis*. The genes specific to *A. salisburgensis* were enriched in functional terms related to protein refolding (GO:0042046, GO:0061077, *HSP20, HSP70, HSP90, CLP, DNAJ/HSP40*), DNA modification (GO:0015074, GO:0005727, GO:0046821, IPR01584, IPR025668, PF13683, GO:0005727, GO:0046821, TCDB 3.A.7), tripartite ATP-independent periplasmic (TRAP), and ATP-binding cassette transporter (IPR010656, IPR000515, TCDB 3.A.1, 2.A.56), as well as chitin degradation (CAZY:GH23) (Figure 1). TRAP transporters were shown to mediate the uptake of compatible solutes in halophilic Proteobacteria such as *Halomonas elongata* [50], while the proteins related to refolding are known to be involved in stress-response [51].

In *A. sclerotialis*, the set of species-specific genes are functionally enriched in terms related to protein folding, heat shock protein 70, and helix-turn-helix (HTH)-binding domain protein (Supplementary Figure S1 and Supplementary Table S1). The helix-turn-helix binding-motif is found in many proteins involved in gene expression regulation [52].

### 3.3. Gene Family Evolution

**Figure 2:**
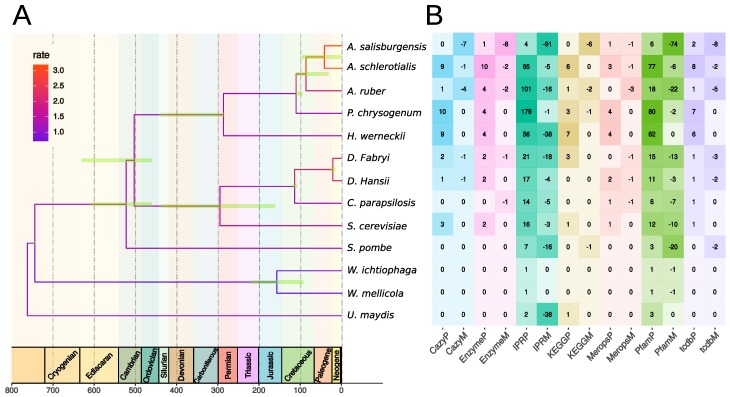
Phylogenomics analyses. (**A**) TimeTree generated from the merged alignment of the one-to-one homologues of the 13 species. The green bars represent the 95% confidence interval. Geologic periods are color-coded and indicated at the bottom of the tree. Evolutionary rate is color encoded. (**B**) CAFE analysis of the expansion and contraction of the annotation elements of KEGG, CAZY, TCDB, Enzyme, and MEROPSfor the 13 species. The suffixes Mand Pafter the annotation categories stand for contraction and expansion, respectively.

A TimeTree of life for the 13 fungi (Table 1) was constructed from the multiple sequence alignments of the proteins shared by all strains and from nine time anchors extracted from the TimeTree database [53] (Figure 2). The results were consistent with the currently-accepted taxonomy [53]. *A. salisburgensis* and *A. sclerotialis* separated approximately 41 MYA (+/−40 MYA), 20 MY before the split between *Debaryomyces fabryi* and *Debaryomyces hansenii* and 40 MY after the split between *A. salisburgensis*, *A. sclerotialis*, and *Aspergillus ruber*. Interestingly, *A. salisburgensis* and *A. sclerotialis* exhibited the largest evolutionary rates, followed by the two *Debaryomyces* species.

This tree was used to estimate the numbers of rapidly-evolving annotation elements with CAFE [39]. The functional annotations used were derived for the carbohydrate enzymes (CAZY) [29], general enzymes from the EggNog annotation [26], InterProScan protein domains (IPR) [54], KEGG pathways [55], proteolytic enzymes (MEROPS) [28], PFAM protein domains [56], and transporters from TCDB [27].

The internal node with the largest numbers of rapidly-evolving annotation families was the one leading to the common ancestor of *Hortaea* and *Aspergillus* (*leotiomyceta*) (Supplementary Table S1). CAFE reported 106 IPR and 105 PFAM rapidly-evolving families. The most significantly expanding families were related to fungal-specific transcription factors (*p*-value 4.75 × 10^−19^ and cytochrome P450 (*p*-value 1.127 × 10^−16^). Cytochrome P450 was also shown to improve salt tolerance in plants [57] and is upregulated under salt stress in the fungus *Piriformosa indica*, a plant endophyte that confers salinity stress tolerance in rice plants [58]. An additional significantly-expanding family was DUF3468 (DUF, domain of unknown function, *p*-value 1.59 × 10^−15^), a protein domain involved in transcription activation of genes related to asexual conidiation and sexual differentiation in *Aspergillus nidulans* and *Aspergillus flavus* [59].

The branch leading to *A. salisburgensis* exhibited the largest contraction of gene families from all species analyzed: 91 IPR, 74 PFAM, 7 CAZY, 8 TCDB, 1 MEROPS, and 8 Enzyme families contracted significantly, which is in line with the reduction in genome size of 27% compared to *A. sclerotialis*. Fungal transcription factors (PF11951) exhibited the largest loss in *A. salisburgensis* (−17). Protein families transporting amino acid and oligopeptide (2A3, −11; IPR002293, −9; 2A18, −5; PF03169, −3), fatty acid (4C1, −7), and iron (2A108, −5) were contracting in *A. salisburgensis*. Interestingly, the amino acid polyamine organocation superfamily (APC) contracted also in *Aspergillus ruber* (2A3, −8). APC is one of the largest families of secondary active transporters, and it is found in all living organisms [60]. Another family depleted in both *A. salisburgensis* and *Aspergillus ruber* is aminoglycoside phosphotransferase, a bacterial antibiotic resistance protein (IPR002575) with a reduction of 15 and 13 members, respectively.

A few annotation families expanded in *A. salisburgensis*. These families were related to the type IV secretory pathway, which exports proteins or DNA-protein complexes out of the cell [61] (3A7 +9), the TRAP-C4 transporter (IPR010656 +6, PF06808 +6), the fungal cellulose binding domain (PF00734, +5), the integrase catalytic core (IPR001584), cutinase (PF01083), sodium:bile-acid symporter/arsenical resistance protein ACR3 (PF01758 +2), and secretory lipase (PF03583, +2). The increase in cellulose degrading ability due to the fungal cellulose binding domain [62] and cutinase [63] is probably related to the environment, a wooden staircase, from which *A. salisburgensis* was isolated. In yeasts, proteins belonging to the PF01758 family confer arsenic resistance by extrusion of sodium arsenate and sodium arsenite [64].

#### Gene Family Enrichment

**Figure 3:**
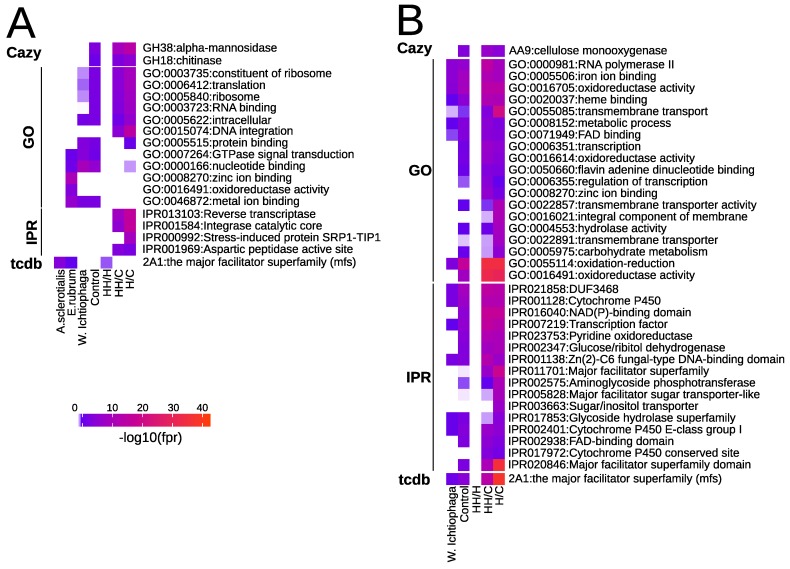
Heat map of the −log(fdr) of the significantly-enriched or -depleted functional annotations in *A. salisburgensis* compared to *A. sclerotialis*, *Aspergillus ruber*, and *Wallemia ichthyophaga*. Depletion/enrichment found in HH vs. H, HH vs. C, and H vs. C is also shown. Only entries with an enrichment/depletion with an fdr < 1e${}^{-5}$ are shown. (**A**) Depletion. (**B**) Enrichment.

The significance of size variations of functional annotation families was studied by comparing species or a group of fungal species (Table 1) with the help of chi-squared tests. The most significant bias was seen for the major facilitator superfamily (MFS) transporters, which showed a peculiar pattern of enrichment. *A. salisburgensis* had significantly more MFS (2A1, IPR020846) transporters than *Wallemia ichthyophaga*, but significantly less than *A. sclerotialis* and *Aspergillus ruber* (Figure 3 and Supplementary Table S1). Further, there were significantly more MFS transporters in halotolerant fungi than in the halophiles (fdr = 0.018). Still, compared to the group of control fungi, both the halophilic (fdr = 3.76e${}^{-15}$) and the halotolerant (fdr = 7.19e${}^{-37}$) fungi were enriched in MFS.

Genes involved in protein translation, such as ribosomes (GO:0003735, GO:0005840), RNA binding (GO:0003723), and reverse transcriptase (GO:013103, GO:0015074), were depleted in halophiles and halotolerant compared to the control group. In contrast, GO terms involved in transcription, such as RNA polymerase II (GO:0000981), transcription (GO:0006531, GO:0006535), and transcription factor (IPR007219, IPR001138), were enriched in both H and HH compared to C. The terms related to cytochrome P450 E-class group I (GO:0016491, GO:0055114, IPR001128, IPR023753, IPR002401, IPR017972) were enriched in both groups of salt-resistant fungi compared to the control group.

### 3.4. Amino Acid Composition

**Figure 4:**
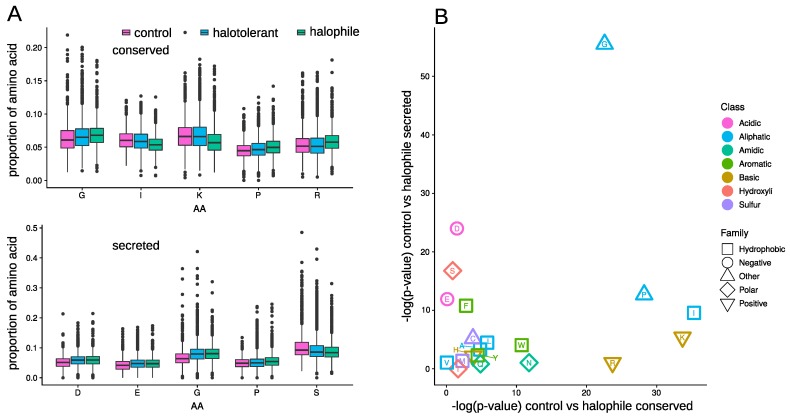
Bias in the amino-acid (AA) distribution between the sets of control, halotolerant, and halophilic fungi. (**A**) Boxplot representing the distribution of AA for the 5 AA with the most significant bias between the control and halophilic group for the set of conserved (top) and secreted proteins (bottom). (**B**) Scatter plot of the −log(p−value) computed with the Wilcoxon test for the AA distribution bias for the set of conserved proteins and the set of secreted proteins.

The protein amino-acid (AA) composition of the C, H, and HH groups for the set of orthologous genes and for the set of exported proteins, i.e., containing a signal-P annotation, was compared (Figure 4 and Supplementary Table S1). With respect to the control group, the conserved proteins in halophilic fungi were significantly enriched in glycine, proline, and arginine and depleted in isoleucine and lysine (Figure 4A). The same bias pattern for those 5 AA was previously reported in the extremely halophilic bacteria *Salinibacter ruber* and *Halomonas elongata* and in the Archaea *Halobacterium salinarum* and *Haloarcula marismortui*, compared to *Escherichia coli* [9]. A low occurrence of lysine and increase of arginine in halophilic proteins is a signature of halophilic microorganisms [65,66]. Psychrophilic organisms, which are similar to halophiles, face reduced water availability and exhibit an increased glycine protein content [67].

Among the set of exported proteins, the acidic AA aspartate and glutamate were overrepresented in halophiles compared to the control group. This is a general characteristic found in many osmotolerant bacteria [65,68]. Similarly, in the three studied HH species, the conserved proteins were enriched in glycine and proline. Finally, the serine depletion was previously reported in halophilic Archaea [66].

### 3.5. Differential Expression between 5% and 20% NaCl

The differential expression of genes between the low and high salt concentrations was studied. Upregulated genes are defined as the set of transcripts that exhibit an increase in expression at 20% salt concentration, while downregulated genes exhibit a reduced expression under high salinity.

#### 3.5.1. *Aspergillus Sclerotialis*

*A. sclerotialis* differentially regulated 2097 genes (1121 up- and 976 downregulated fdr < 0.05, $|log_{2}FC|$ > 1) between both salt concentrations. The most strongly upregulated gene (x3536) belonged to the major facilitator superfamily (MFS) transporter, which are membrane transport proteins that facilitate movement of small solutes across cell membranes in response to osmotic gradients [69]. Among the 96 genes upregulated by a factor larger than 50 in the halotolerant fungus, 14 genes were related to transmembrane transport (Supplementary Table S1). Cerato-ulmin hydrophobin, a parasitic fitness factor of the agents of Dutch elm disease [70], was upregulated by a factor of 2641. The regulation of this hydrophobin might indicate a role in salt resistance, as previously reported in *Wallemia ichthyophaga* [15]. Given the fact that the halotolerant strain is a dog pathogen, this hydrophobin might be involved in the pathogenicity of the strain [71].

The C-terminal dimerization domain found in transposases of elements belonging to the activator superfamily was increased by a factor 280 under high salt conditions, indicating that DNA modification might take place. A similar feature was seen in sunflower exposed to salt and drought stress [72]. Transcriptional regulators are also found among the most upregulated genes [73]. A gene involved in protein neddylation [74] had its expression increased by a factor of 1571. Inositol monophosphatase, which is upregulated by a factor of 571, is involved in the phosphatidylinositol signaling pathway and was shown to increase Na${}^{+}$ resistance in yeast [75]. An F-box domain-containing protein was upregulated by a factor of 286 upon salt-induced stress, which is in line with previous reports in *Schizosaccharomyces pombe* [76]. Interestingly, two genes involved in the synthesis of antibiotics, Acyl-CoA 6-aminopenicillanic-acid-acyltransferase and pristinamycin IIA synthase subunit A, were upregulated by a factor of 369 and 338, respectively.

Among the genes downregulated by a factor larger than 50, eight were related to transporters, and one was related to SAM-dependent methyltransferase. Chorismatase-degrading enzymes were downregulated, in agreement with the salinity stress experiment in *Carthamus tinctorius* [77], where chorismatase synthase was upregulated upon salinity stress, indicating that chorismate plays a role in salt resistance. Two cupredoxin were downregulated by a factor of 247. A reverse transcriptase and a integrase were downregulated 95- and 129-times, respectively, while one Zn${}_{2}$-Cys6 transcription factor was downregulated by a factor of 124.

**Figure 5:**
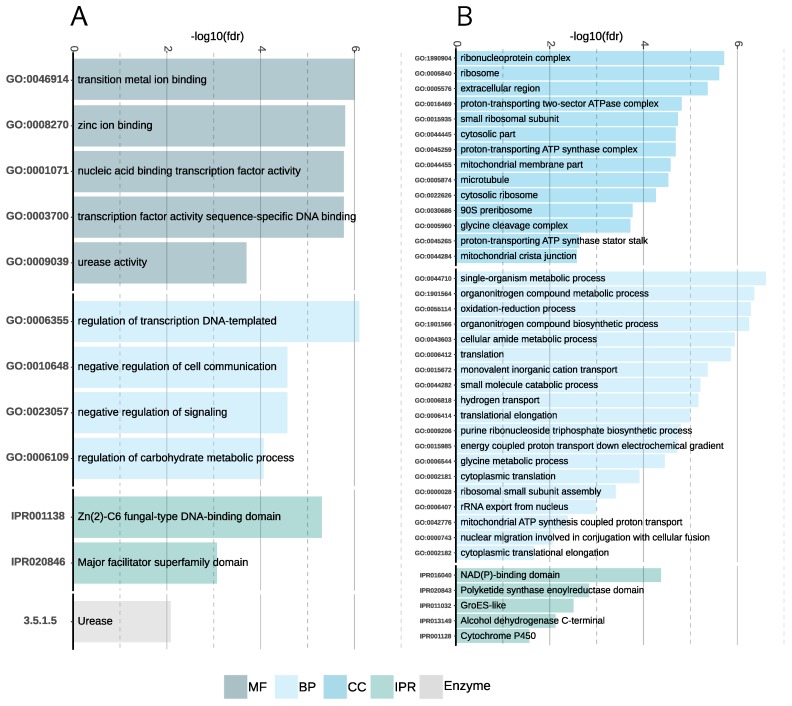
Bar plots showing overrepresented functional annotations in the set of regulated genes in *A. sclerotialis*. (**A**) Enriched functional terms in the set of downregulated genes. (**B**) Enriched functional terms in the set of upregulated genes.

The functional enrichment analysis for the downregulated genes indicates that transcriptional factors, MFS transporters, and urease activity (Enzyme 3.5.1.5 fdr = 0.0083, GO:0009039 fdr = 3.15$e^{-4}$) were negatively impacted by the high salt concentration (Supplementary Table S1 and Figure 5A). It was previously shown that a depletion of urease improves salt resistance in *Arabidopsis thaliana* [78].

For the set of upregulated genes (Figure 5B), an enrichment in translation, glycine metabolism, oxidation-reduction process, mitochondrial ATP synthesis, and microtubule was seen. Osmotic stress was previously shown to increase the free glycine concentration in wheat transiently [79]. The five upregulated genes related to microtubules were two tubulins (jg1358, jg9339), one kinesin (jg11911), one dynein (jg2827), and one cytoskeleton-associated protein (jg228). These genes are involved in the transportation of vacuoles and organelles along the microtubules [80].

#### 3.5.2. *A. salisburgensis*

In *A. salisburgensis*, 305 and 306 gene were up- and down-regulated, respectively. The most upregulated gene (x9013) was a peptidase S8 subtilisin protein with putative keratinolytic activity. Although the biological functions of most fungal subtilases are not yet described, some attempts led to the assumption that subtilisins (S8) could be related to a saprotrophic lifestyle [81]. More recently, a study showed striking correlations for subtilisins’ (S8) expansion in pathogenic and soil-/dung-inhabiting fungi [82]. Subtilases have further been reported to be involved in drought and salt resistance mechanisms in plants [83]. An example is the *Arabidopsis thaliana* subtilase ATSBT6.1, which is associated with the unfolded protein response on salt stress through the cleavage of an ER-resident type II membrane protein (BZIP28). The cleavage of this protein is essential for the activation of genes associated with the salt stress response [84].

Among the proteins with at least 50-fold upregulation, eight proteins were glycoside hydrolases, three were involved in oxido-reduction processes, four exhibited a cupin-like domain, and two were involved in post-translational modification (Supplementary Table S1). Among the set of genes downregulated at least 50-fold, four ribitol dehydrogenases, seven transporters, two cyclic amino-acid related enzymes, two transcription factors, and two alcohol dehydrogenase were found.

**Figure 6:**
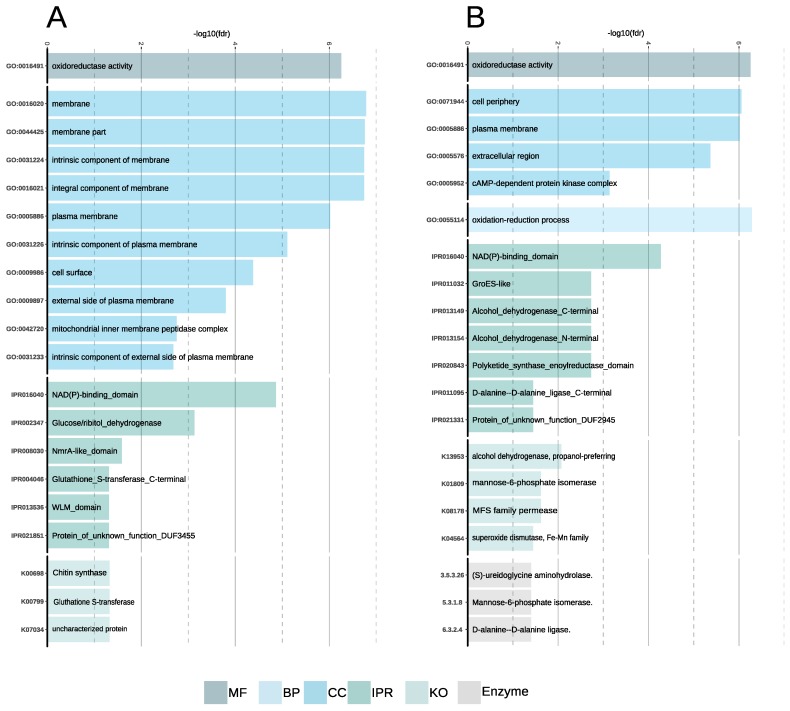
Bar plots showing overrepresented functional annotations in the set of regulated genes in *A. salisburgensis*. (**A**) Enriched functional terms in the set of downregulated genes. (**B**) Enriched functional terms in the set of upregulated genes.

Functional enrichment analyses of the downregulated genes indicated that the expression of membrane-located proteins, especially serine-/threonine-rich proteins, were downregulated (Figure 6A and Supplementary Table S1). Similarly, the gene coding for glutathione S-transferase, which was previously shown to play a negative role in salt stress tolerance in *A. thaliana* [85], was downregulated under high salinity condition. Chitin synthase genes were downregulated, which is in line with proteomic results in yeast [86].

The set of upregulated genes covered a broader spectrum of functions than that of the downregulated genes. Genes located at the cell periphery and extracellular region were overrepresented in the set of upregulated genes (Figure 6B and Supplementary Table S1). The over-representation of functional terms related to cell wall production like the d-alanine–d-alanine ligase and mannose-6-phosphate isomerase might indicate that cell wall damages are happening under high salinity [87]. Enrichment of superoxide-dismutase (K04564), which is an enzyme involved in the catalysis of superoxide, is a known marker of oxidative and salt stress [88]. Finally, the over-representation of MFS transporters is a paramount osmotic stress response, as these transporters are transporting small molecules in response to chemiosmotic ion gradients [69].

#### 3.5.3. Comparative Transcriptomics

Difference and similarities in expression of genes found in both studied fungal strains were assessed. A total of 84 genes was one-to-one homologous in both strains and was significantly regulated. Genes upregulated in the halophilic and halotolerant species were related to polyketide synthase, chloroperoxidase, haloacid dehalogenase, fructose-biphosphate aldolase, copper transport, and pectinolytic enzyme (Supplementary Table S1). Chloroperoxidases were shown to be potential chlorinators of lignin in plant materials and to contribute to lignin degradation [89]. This is similar to the use of chlorine for wood pulp delignification [90]. In the fungi *Fusarium fujikuroi* and *Neurospora crassa*, genes belonging to the haloacid dehalogenase family were shown to be involved in the osmotic stress and glycerol metabolism [91]. Conserved genes downregulated in both species are related to amino acid transport, trehalose-phosphatase, thiolase, and the p53 transcription factor.

Genes solely upregulated in *A. sclerotialis* were related to oxido-reductive processes, zinc transport, and polyketide synthase. In contrast, genes upregulated in the halophilic fungus and downregulated in the halotolerant fungus were related to the cation/H^+^ exchanger, threonine/serine exporter, voltage-dependent channel, intradiol cleavage, the putative sensor protein SUR7/RIM9, which is a pH-response regulator protein, and the isochorismatase, a gene involved in salicylic acid metabolism. The upregulation of the last gene only in the halophilic strain may indicate an adaptive advantage to survive permanently in a hypersaline environment. Salicylic acid (SA) is a natural phenolic compound known to control many physiological and biochemical functions in plants such as growth, development, and responses to abiotic stresses [92]. Some studies have shown that SA plays a role in the response to salinity in plants and that its exogenous application improves tolerance to salt stress in several species [93].

### 3.6. Comparison to Fungal Osmoadaptation and Osmoregulation

The literature on osmoadaptation and osmoregulation in fungi was compared to the genome content and transcription patterns of *A. sclerotialis* and *A. salisburgensis*.

#### 3.6.1. Osmosensing

All members of both branches of the high osmolarity signaling pathway were found in *A. salisburgensis* and *A. sclerotialis* [94,95]. Histidine kinase 7A/B [96] from *Hortaea werneckii*, the mitogen activated protein kinase (KSS1) [97] from *Saccharomyces cerevisiae*, both involved in signal transduction, and the cell wall integrity sensor (MID2) [98] were found in the halotolerant, but not in the halophile genome. Interestingly, the irritation of the cell wall and membrane by shrinking triggers induction of the HOG signaling pathway (e.g., in *S. cerevisiae* [99,100]). Inversely, *A. salisburgensis* has a homologue of the tyrosine-protein phosphatase 3 (PTP3), which is involved in the repression of the *HOG1* gene [101] (Supplementary Table S1).

Protein kinase A (PKA1, TPK1), an enzyme generally involved in the regulation of the glycogen, sugar, and lipid metabolism that negatively regulates the transcription factors (MSN2/MSN4) [102], was upregulated six-fold in *A. salisburgensis*, but downregulated by a factor of three in *A. sclerotialis*. *TOR1*, another gene that negatively regulates the MSN2/MSN4 transcription factors [102], was also downregulated by a factor five in *A. sclerotialis*, but not in *A. salisburgensis*. The PBS2 MAP kinase kinase, which is involved in the HOG pathway, and GSP2, a histidine kinase involved in the nuclear import of HOG1 [103], were upregulated 5.5- and 4.8-fold at a high salt concentration in *A. sclerotialis*, but not in *A. salisburgensis*. NIK1, a histidine kinase involved in the SLN1 branch of the HOG pathway [104], was downregulated nine-fold in *A. sclerotialis*.

#### 3.6.2. Ion Homeostasis

The genome of *A. salisburgensis* contains most of the ion transporters reported to be involved in ion trafficking during osmotic stress in *Saccharomyces cerevisiae* and *Hortaea werneckii*, with the exception of the high affinity potassium transporter (HAK1) [104], the potassium antiporter (KHA1) [15], and the outward-rectifier potassium channel TOK1 [15]. In *A. sclerotialis*, the endosomal/prevacuolar sodium/hydrogen exchanger (NHX1) and HAK1 are missing. TOK1 and the mitochondrial exchanger system, while present in the halotolerant strain, were downregulated four-fold at 20% NaCl in this strain. PAM1, a proton ATPase involved in salt resistance in *Debaryomyces hansenii* [105], was upregulated by a factor of 13 in *A. sclerotialis*. Similarly, NHA1, the Na^+^/H^+^ antiporter, was upregulated 6.3-fold under high salt concentration. In the obligate halophile, ENA1, a P-type ATPase sodium pump involved in Na^+^ and Li^+^ efflux [106] was upregulated by a factor of 39.

#### 3.6.3. Cellular Respiration

In *A. sclerotialis*, seven genes annotated as involved in respiration (GO:0045333) were upregulated under high salt concentration. The electron carrier protein CYC1 was upregulated by a factor of 4.62; the cytochrome BC1 complex subunit 7 (QCR7) expression increased by a factor of 3.94; and the cytochrome c oxidase subunit VIa (COX13) was induced by a factor of 3.63. Further, an ADP/ATP carrier protein (AAC) was upregulated by a factor of 6.14. Mitochondrial isocitrate dehydrogenase (IDH1), malate dehydrogenase (MDH1), and mitochondrial trans-2-enoyl-CoA reductase were upregulated 4.82-, 12.82-, and 4.62-fold, respectively. This strongly indicates that the ATP production is increased during salt stress in the halotolerant strain. In contrast, no genes related to cellular respiration were significantly regulated in *A. salisburgensis*.

#### 3.6.4. Stress Response

*A. sclerotialis* had a stress response more pronounced than *A. salisburgensis*, both in terms of the number of regulated genes, as well as in the regulation intensity. Markers of oxidative stress response were strongly upregulated in *A. sclerotialis* at a high salt concentration, which is in line with the increased respiratory process seen in the halotolerant strain. The mitochondrial superoxide dismutase (SOD2), an important antioxidant defense, catalyzes the dismutation of superoxide O${}_{2}^{-}$, a mitochondrial byproduct of respiration, into oxygen and hydrogen peroxide. This gene was upregulated by a factor of 8.3 in *A. sclerotialis* at high salinity. H_2_O_2_ is then reduced to H_2_O by peroxiredoxin (PRX1) using electrons from the reduced form of thioredoxin (TRX) [107]. In *A. salisburgensis*, TRX was upregulated by a factor of 3.6 at a 20% salt concentration. Two mitochondrial PRX1 were upregulated by 9.5- and 6.5-fold, respectively. Further, the nuclear thioredoxin peroxidase DOT5 and the AHP1 peroxiredoxin were upregulated by a factor 4.8 and 5.9, respectively. H_2_O_2_ can also be scavenged through the glutathione system with the help of glutathione redoxins (GRX) [107]. A homologue to GRX1 and GRX2 and a homologue to GRX3 and GRX4 were upregulated 6.31 and 4.3 in *A. sclerotialis*, respectively. Two catalase paralogues (CTT1), an enzyme that is involved in peroxide scavenging, were upregulated by a factor of 10.1 and 6.9, respectively. The homologue to MCA1, a regulator of apoptosis upon H_2_O_2_ and clearance of insoluble protein aggregates, was upregulated by a factor of 3.71 [108,109]. Additional studies have shown that oxidative stress originating from intensive mitochondrial respiration [110] can pose a further threat to the survival of aerobic microorganisms living in high-salinity environments. Therefore, this stress can be one of the limiting factors for growth in such environments. In the halotolerant strain *Hortaea werneckii*, the levels of ROS degradation and resistance determine the upper limit of salt tolerance [111]. The peptidyl-prolyl cis/trans-isomerase, a gene involved in protein folding [112], underwent an upregulation under salt stress by a factor of 3.43 in the halotolerant strain.

In *A. salisburgensis*, the oxidative stress response was moderate. Similar to the halotolerant strain, CTT1 was upregulated by a factor of 8.8. Methionine sulfoxide reductase (MXR2), a gene protecting against methionine oxidation by catalyzing thio-dependent reduction of oxidized methionine residues [113], saw a 5.29-fold increase in transcription. Interestingly, the obligate halophile reduced the production of superoxide radicals by downregulating NADPH oxidase and the NADPH oxidase regulator by a factor five and 10, respectively. Beyond genes involved in oxidative stress response, *A. salisburgensis* upregulated HSP12, a chaperone regulated in osmotic stress conditions in *Saccharomyces cerevisiae*, by a factor of five [114].

#### 3.6.5. Cell Interface

In the extracellular region, *A. salisburgensis* strongly upregulated the subtilisin peptidase (S8 x8964), two cellulose degradation enzymes (GH51, x62), a pectinolytic enzyme (EC:4.2.2.2 x23), and a cellulase (GH5 x22.62). Together with the upregulation of a chloroperoxidase, which might be involved in the degradation of lignin, and the environment where this fungus has been isolated, i.e., a wooden staircase in a salt mine, it seems reasonable to assume that salt triggers a cellulolytic response in the obligate halophile. *A. sclerotialis* strongly upregulated a hydrophobin (PF06766 x2646).

In the cell wall of the halotolerant fungus, a beta glucosidase involved in cell wall remodeling (UTH1) [115] was upregulated by a factor of 17, while a membrane-bound HSP70 involved in selective cation trafficking was upregulated by a factor of 6.33 [116]. In the halophilic fungus, the expression of a lysophospholipase increased 18-fold. This enzyme is involved in phospholipid degradation and was previously reported to be upregulated in the micro-algae *Dunaliella salina* under high salt concentration [117].

At the membrane level, besides the previously discussed transporters, *A. sclerotialis* increased the expression of a putative ammonia transporter (PKC1 x22.31), a dipeptidase (DPE1, x9.91), the SUR7 membrane protein involved in membrane organization and cell wall stress [118] (x7.78), a sulfate permease (SULP x6.77), and a cellulolytic enzyme (EggNog: 0PG84 x11.15). The halophile strain increased the transcription of two guanine nucleotide proteins (G protein) [119] (ion-translocating rhodopsin x10.63, RHEB x4.00) and one G protein-coupled receptor-like GPR1 (x12.46), a gene involved in endocytosis, the eisosome component PIL1/LSP1 (x32.11) [120], and two SUR7 membrane proteins (x4.37, x25.73).

#### 3.6.6. Compatible Solute Management

Known genes involved in compatible solutes’ management from halotolerant and obligate halophilic fungi were reviewed. Compatible solutes are known to be accumulated or synthesized during changed osmotic conditions. One of these is D-mannitol, which can be synthesized by a reduction of fructose, catalyzed by NADP-dependent mannitol dehydrogenases [13]. In *A. salisburgensis*, two homologues of NADP-dependent mannitol dehydrogenases were found in the genome, but were not regulated in high salt concentration, while one homologue was found in *A. sclerotialis* and was upregulated by a factor of 29. Still, *A. salisburgensis* upregulated GRE3, an aldole reductase involved in polyol metabolism [114], by a factor of 28.

Three homologous genes of STL1, a glycerol/proton symporter of the plasma membrane, were found in *A. salisburgensis* and one in *A. sclerotialis*. In comparison, four homologues were found in the genome of the obligate halophilic fungus *Wallemia ichthyophaga* [15]. In *A. salisburgensis*, one copy of the genes was upregulated by a factor of eight, and in *A. sclerotialis*, STL1 was upregulated by a factor of 91. In *Saccharomyces cerevisiae*, it was reported to be upregulated during hyperosmotic shock [121]. The aquaglyceroporin channel FPS1, which is opened in hypoosmotic conditions to release glycerol [122], did not receive any regulation in *A. salisburgensis* and was downregulated in *A. sclerotialis* by a factor of 30.

Glycine betaine is an osmoprotectant found in plant, animals, bacteria, and fungi and is involved in reactive oxygen scavenging [123]. In *Aspergillus fumigatus*, glycine betaine is produced by converting first choline to betaine aldehyde with a choline oxidase. Betaine aldehyde or its rapidly-forming hydrated equivalent gem-diol-choline are then converted to glycine betaine with the help of betaine aldehyde dehydrogenase or choline oxidase, respectively [123]. In *A. salisburgensis*, two choline oxidases and one betaine aldehyde dehydrogenase were found, while in *A. sclerotialis*, two copies and one copy were found, respectively. While *A. salisburgensis* did not significantly regulate these enzymes, *A. sclerotialis* increased the transcription of betaine aldehyde dehydrogenase by a factor of 4.5 in the high salinity condition.

Genes involved in polyols metabolism were also strongly upregulated. Polyols (also called sugar alcohols) compensate for differences between the extracellular and intracellular water potential without affecting the integrity and function of proteins [124]. In *A. sclerotialis*, gene jg11062 belongs to the same group as the l-arabitol 4-dehydrogenase from *Aspergillus fumigatus* and was upregulated by a factor of 1063 at high salt concentration (Supplementary Table S1). A sorbitol dehydrogenase and a ribitol dehydrogenase were upregulated by a factor of 62 and 67, respectively. The important role of polyols for the resistance of extremotolerant fungi under osmotic stress was already described by Sterflinger [125]. However, this general protection strategy is widespread also in bacteria and Archaea [126].

In the halotolerant strain, four SAM-dependent methyltransferases showed an increase in transcription by a factor of 455, 218, 205, and 52, respectively, similar to previous reports in algae [127], where sequence homologues of the SAM-dependent methyltransferase are involved in the production of the compatible solute homoserine betaine [128]. Glutamine synthetase was upregulated by a factor of 181, indicating that *A. sclerotialis* might use glutamine as an osmolyte, something previously reported for *Halobacillus halophilus* [129].

## 4. Conclusions

In this work, the genomic adaptation and gene regulation of the obligate halophile *A. salisburgensis*, a fungus isolated from a wooden staircase in a salt mine, were studied by employing comparative genomics and transcriptomics approaches. The halotolerant relative *A. sclerotialis*, a pathogen, was used to gain insight into the environment-specific osmoadaptation and regulation of the obligate halophile.

On the genomic level, *A. salisburgensis* exhibited a 27% decrease in genome size and gene content compared to *A. sclerotialis*. Considering the unique, extremely stable niche where the obligate halophile has been found, it can be hypothesized that the fungus optimized its genome content by dumping genes unnecessary for its survival, a known phenomenon in bacteria [130]. Niche adaptation is further seen in the enrichment of genes involved in cellulose degradation. Halophile-specific depletion of MFS transporters previously reported in *Wallemia ichthyophaga* and *Aspergillus ruber* [10,15] was also found in *A. salisburgensis* and might therefore be a strategy to survive in the high saline environment. This study further confirmed the specific amino-acid enrichment and depletion patterns found in other halophilic species compared to the control species.

The fact that the obligate halophile regulated three-times fewer genes than the halotolerant strain between both salinities further underlines the adaptation of the former to high salt concentration. Besides the regulation of a few transporters like ENA1, STL1, a hydrophobin, and an aldol-reductase, a gene reported to be involved in the production of compatible solutes, there was almost no sign revealing that the obligate halophile was under stress at a 20% salt concentration. Instead, the cellulolytic activity was an indication that the high salt concentration was beneficial to *A. salisburgensis*, as it triggered the expression of a battery of wood-degrading enzymes. Among them, the chloroperoxidase was making use of the chloride ion to chlorinate lignin and improved its degradation.

In contrast, the halotolerant strain exhibited both an osmotic- and oxidative-stress response. The link between both stresses might probably be found in the respiration processes. In fact, it is probable that under high salinity, homeostatic regulation requires an increased supply of ATP, which is produced mainly from respiration. At the transcriptome level, the increased respiration is seen in the upregulation of genes involved in the electron transport chain and the citrate cycle. ROS originating from the respiration induced an oxidative stress response, as can be seen from the increased transcription of genes involved in oxidative-stress, such as superoxide dismutase, thioredoxin peroxidases, glutathione oxidoreductases, and catalases. *A. sclerotialis* further upregulated a hydrophobin, a family of proteins that plays a role in salt resistance in *Wallemia ichthyophaga* and in pathogenicity. 

References

## Figures and Tables

**Table 1:** List of genomes compared in this study. For each species, the name, the range of salt tolerance, the type of tolerance, and reference are listed. H: halotolerant, HH: halophile, C: control.

Species	Salt Concentration	Type	Reference
*A. salisburgensis*	5–30%,optimal at 20%	HH	This publication
*Aspergillus ruber*	>10%, optimal at 18%	HH	[10]
*Wallemia ichthyophaga*	>8%, optimal 18%	HH	[13]
*A. sclerotialis*	0–20%, optimal at 10%	H	This publication
*Hortaea werneckii*	0–32%, optimal 3–9%	H	[15]
*Penicillium chrysogenum*	0–18%, optimal at 10%	H	[14]
*Candida parapsilosis*	0–12%, optimal 0 %	H	[16]
*Debaryomyces fabryi*	0–16%, optimal 0 %	H	[17]
*Debaryomyces hansenii*	0–24%, optimal 0 %	H	[17]
*Wallemia mellicola*	0–27%, optimal 0 %	H	[47]
*Saccharomyces cerevisiae*	0–8%, optimal 0 %	C	[48]
*Schizosaccharomyces pombe*	0–5%, optimal 0 %	C	[48]
*Ustilago maydis*	0–7%, optimal 0 %	C	[18,49]

## Supplementary Materials

The supplementary table and the supplementary figure are available online at https://www.mdpi.com/2073-4425/10/5/381/s1.
