# Supplementary figures and images for: Back to the Salt Mines: Genome and Transcriptome Comparisons of the Halophilic Fungus Aspergillus salisburgensis and Its Halotolerant Relative Aspergillus sclerotialis
Source: Genes (Basel). 2019 May 20;10(5):381. doi: 10.3390/genes10050381 (PMC6563132; doi:10.3390/genes10050381)

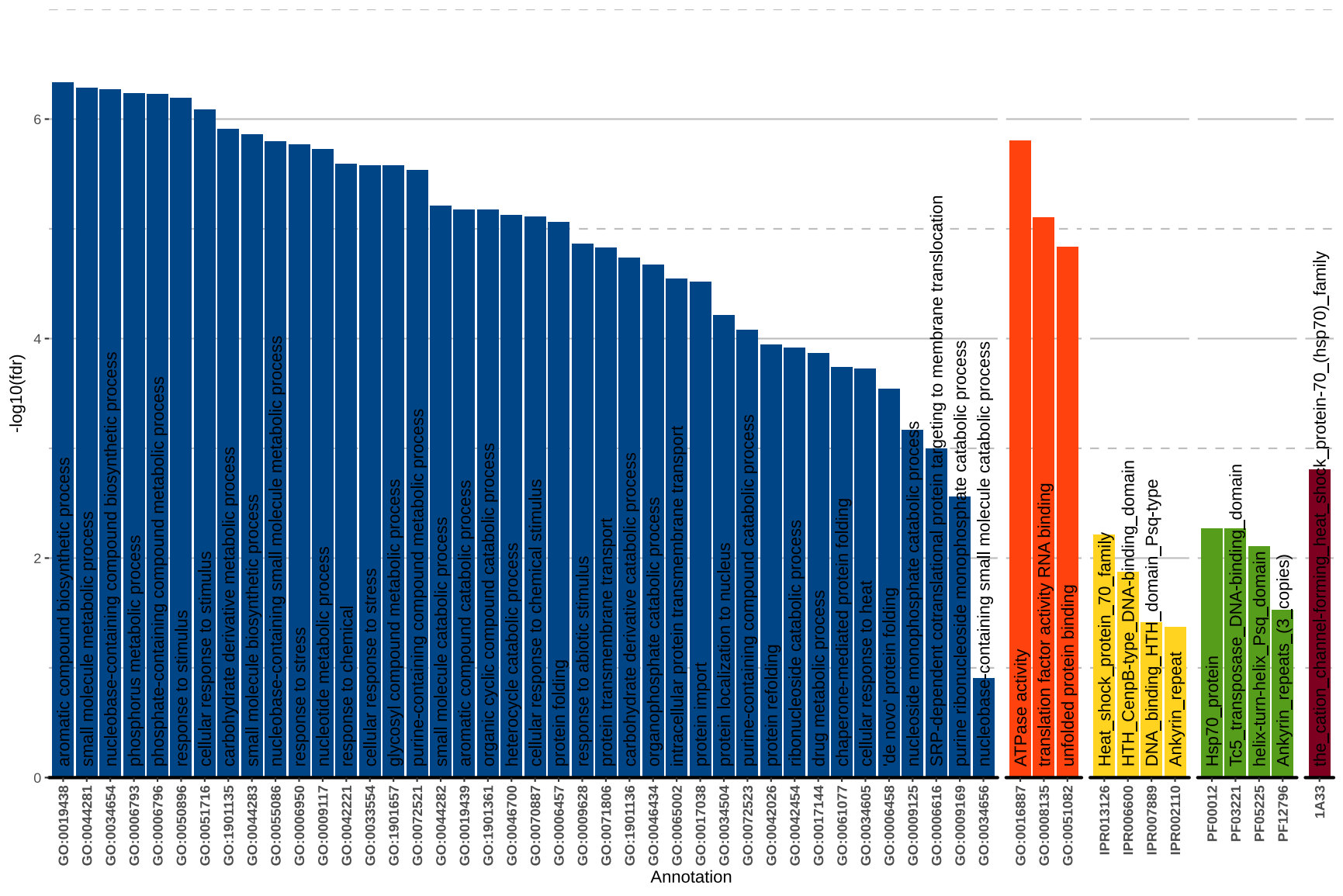

Supplement: Supplementary file 1 [file genes-10-00381-s001.zip › toPlot.specificToPhiScl.pdf]
